# Identification of Plasmatic MicroRNA-206 as New Predictor of Early Recurrence of Atrial Fibrillation After Catheter Ablation Using Next-generation Sequencing

**DOI:** 10.1007/s40291-024-00698-x

**Published:** 2024-03-08

**Authors:** Filip Šustr, Táňa Macháčková, Martin Pešl, Jana Svačinova, Karolína Trachtová, Zdeněk Stárek, Bohuslav Kianička, Ondřej Slabý, Jan Novák

**Affiliations:** 1 2nd Department of Internal Medicine, St. Anne’s University Hospital in Brno and Faculty of Medicine of Masaryk University, Pekařská 53, 602 00 Brno, Czech Republic; 2 Department of Physiology, Faculty of Medicine, Masaryk University, Brno, Czech Republic; 3 Ondrej Slaby Joint Research Group, Central European Institute of Technology and Department of Biology of Faculty of Medicine, Masaryk University, Brno, Czech Republic; 4 1st Department of Internal Medicine, Cardioangiology, St. Anne’s University Hospital in Brno and Faculty of Medicine of Masaryk University, Brno, Czech Republic

## Abstract

**Background:**

Catheter ablation (CA) of atrial fibrillation (AF) is indicated in patients with recurrent and symptomatic AF episodes. Despite the strict inclusion/exclusion criteria, AF recurrence after CA remains high. Identification of a novel biomarker that would predict AF recurrence would help to stratify the patients. The aim of the study was to seek novel biomarkers among the plasmatic microRNAs (miRNAs, miRs).

**Methods:**

A prospective monocentric study was conducted. A total of 49 consecutive AF patients indicated for CA were included. Blood sampling was performed prior to CA. RNA was isolated from plasma using commercial kits. In the exploration phase, small RNA sequencing was performed in ten AF patients (five with and five without AF recurrence) using Illumina instrument. In the validation phase, levels of selected miRNAs were determined using quantitative reverse transcription polymerase chain reaction (qRT-PCR) in all participants.

**Results:**

Altogether, 22 miRNAs were identified as altered between the groups by next-generation sequencing (using the DESeq2 algorithm). Using qRT-PCR, levels of the five most altered miRNAs (miR-190b/206/326/505-5p/1296-5p) were verified in the whole cohort. Plasma levels of hsa-miR-206 were significantly higher in patients with early (within 6 months) AF recurrence and showed an increase of risk recurrence,2.65 times by every increase in its level by 1 unit in the binary logistic regression.

**Conclusion:**

We have identified a set of 22 plasmatic miRNAs that differ between the patients with and without AF recurrence after CA and confirmed hsa-miR-206 as a novel miRNA associated with early AF recurrence. Results shall be verified in a larger independent cohort.

**Graphical Abstract:**

**Figure:**
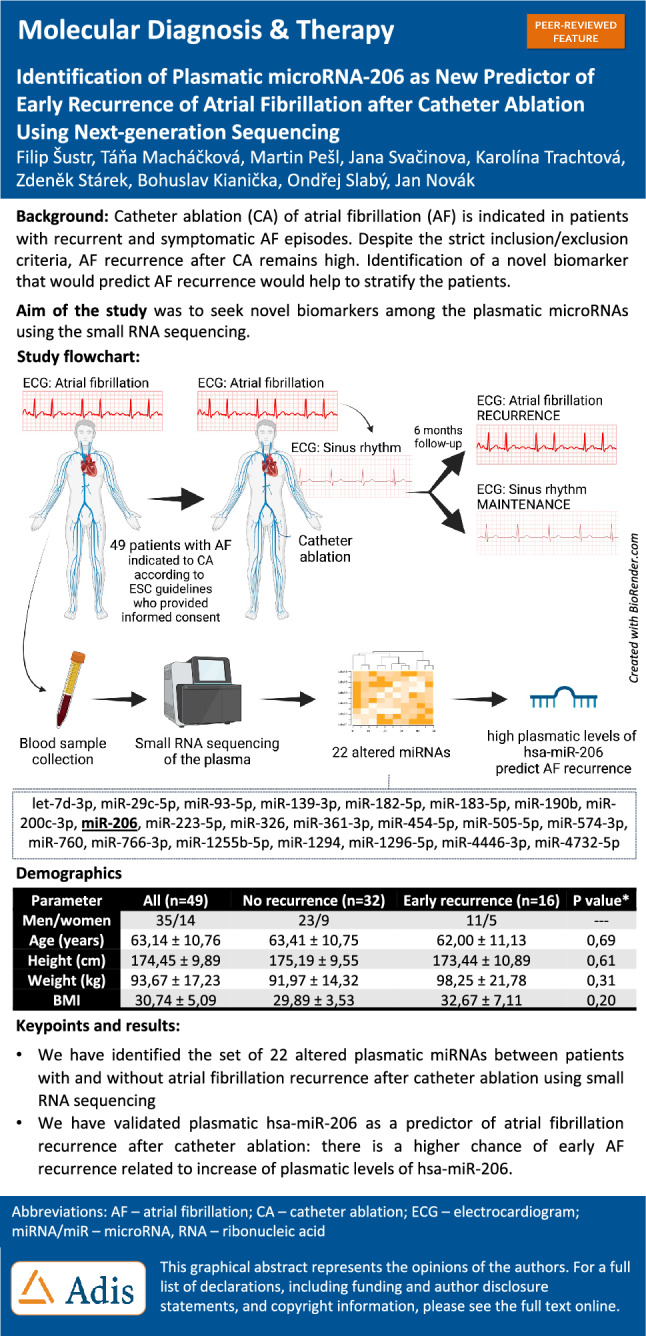
Graphical abstract summarizing the study design and results

**Supplementary Information:**

The online version contains supplementary material available at 10.1007/s40291-024-00698-x.

## Key Points

**Table:** 

An easily accessible biomarker of atrial fibrillation recurrence after catheter ablation is still lacking.
We have discovered a set of 22 altered plasmatic microRNAs between patients with and without atrial fibrillation recurrence after catheter ablation using small RNA sequencing.
We have validated plasmatic hsa-miR-206 as a predictor of atrial fibrillation recurrence after catheter ablation.

## Introduction

Atrial fibrillation (AF) is the most common supraventricular arrhythmia, with an estimated prevalence of 37,574 million cases in the world (approximately 0.51% of the world population), and it continues to grow [1]. Predictions suggest that Europe will have a rapid, more than two times, increase of AF patients in the next decades [1–3], mainly due to population aging [4]. AF represents a major cardiovascular risk factor, with its complications and the high economic burden for healthcare (up to 2.6% of healthcare expenditure in Europe) [5–7]. Standard AF treatment is initiated using antiarrhythmic drugs to reach rate or rhythm control, together with anticoagulation therapy to avoid embolic episodes [8]. In patients with low to no response to the treatment or in patients with recurrent and symptomatic episodes of AF, fulfilling the indication criteria determined by the current guidelines [8] , catheter ablation (CA) is indicated, mostly by pulmonary vein isolation [9].

Despite the defined criteria, there is still a high rate of recurrence of AF after the procedure, ranging between 20 and 61%, dependent on the CA strategy and number of performed procedures [10,11]. The risk of AF recurrence after CA can be estimated by score systems, namely the APPLE Score containing age, type of AF, impairment of estimated glomerular filtration rate (eGFR), diameter of left atrium (LA) and ejection fraction (EF) [12]. A similar score system named CAAP-AF includes presence of coronary artery disease, LA diameter, age, type of AF, failure of antiarrhythmic therapy and sex [13]. Concomitant diseases, mainly indicators of metabolic syndrome (obesity, arterial hypertension, diabetes mellitus and dyslipidaemia), are also mentioned as recurrence risk factors in the literature [14]. Besides the clinical parameters, a recent meta-analysis summarized several laboratory parameters associated with the AF recurrence after CA as natriuretic peptides (mainly N-terminal prohormone of B-type natriuretic peptide [NT-pro-BNP] and B-type natriuretic peptide [BNP]), markers of fibrosis (galectin-3, transforming growth factor-β), inflammatory pathway markers (C-reactive protein [CRP], tumour necrosis factor-α, interleukin-6, white blood cell count), lipid profile markers and other parameters (creatinine, eGFR, troponin I, uric acid) [15]. However a specific, sensitive and easily obtainable biomarker that would predict AF recurrence after CA is still missing. The potential biomarkers of AF recurrence after CA may be identified among the non-coding RNAs, specifically microRNAs (miRNAs, miRs).

miRNAs are small non-coding RNAs of an average length of 22 nucleotides. miRNAs play a crucial role in the post-transcriptional regulation of gene expression via RNA interference by binding to target messenger RNAs (mRNAs). miRNAs can cause blockade or decay of these mRNAs and thereby change the final levels of encoded proteins. Besides this intracellular role, miRNAs can also be found in the extracellular space (e.g. in blood), where some of them act as messengers in intercellular communications or as markers of organ damage (as some miRNAs are tissue specific and can be released into circulation after the tissue damage) [16]. The association of several specific miRNAs and onset or recurrence of AF was repeatedly observed. Some miRNAs are connected to the electrical (e.g. miR-1, miR-25, miR-26, miR-133, miR-328) and structural (e.g. miR-21, miR-29b, miR-30, miR-150, miR-409, miR-590) remodelling of the myocardium during AF [17]. Some miRNAs (miR-103a, miR-107, miR-320d, miR-486) are increased in the plasma/serum of AF patients compared to controls; the others are decreased between these two groups (miR-21, miR-29b, miR-150, miR-328). Even alteration between patients with persistent versus paroxysmal AF (e.g. miR-21, miR-150) can be observed, as was recently reviewed by our group [18]. However, our knowledge regarding circulating miRNAs predictive of AF recurrence after CA or the whole spectrum of miRNAs present in AF patients’ plasma is limited to pilot studies [19]; thus within the current study, we aimed to identify the potential plasmatic miRNAs predicting AF recurrence after CA using small RNA sequencing.

## Materials and Methods

### Study Design

A prospective monocentric study was conducted. The study was approved by the ethics committee of the St. Anne´s University Hospital in Brno (approval 3G/2016) and was performed according to the Declaration of Helsinki.

In a period of 3 years (March 2016–March 2019), 49 consecutive patients with AF indicated for CA according to current guidelines [8] and willing to participate in the study were enrolled. Exclusion criteria included patients under the age of 18 and those with structural pathology of the heart or severe valvular disease, thrombus in LA or its appendage, hyperthyroidism or electrolyte imbalance, acute coronary syndrome or stroke in the last 6 months, major surgical procedure or trauma in the last 2 months, cancer, an immune system disorder, and liver or kidney failure.

Enrolment of the patients took place during their hospitalization at the 1st Department of Internal Medicine: Cardio-Angiology of St. Anne´s University Hospital. After admission, all eligible participants fulfilling the abovementioned criteria were offered the opportunity to participate in the study. Those who agreed provided their written informed consent. After signing the informed consent, patients had their past medical history collected, and all patients underwent CA using St. Jude Medical, Inc. (SJM) EnSite Velocity or SJM EnSite Precision Systems. Performance of the CA and choice of system were at the discretion of the caring cardiologist and were not affected by participation in the study. Data about the CA were collected for the study purposes.

After the CA, patients were invited for the follow-up visit after 6 months. During this visit, early AF recurrence was evaluated. Recurrence was considered to be any episode of AF in the electrocardiogram (ECG) Holter record, need of re-ablation during the follow-up period, or any lasting type of AF confirmed by ECG during the follow-up period. Findings of other types of arrhythmias (e.g. atrial flutter or more frequent occurrence of extrasystoles) were not considered as AF recurrence.

Due to the coronavirus disease 2019 (COVID-19) outbreak, another follow-up control, which was planned for after 2 years since initial CA, was performed remotely to document late AF recurrence. During this visit, either the patient or their treating cardiologist was contacted by phone by the study team. Three main questions were asked: Did you (your patient) undergo another CA or cardioversion because of AF recurrence during the last 2 years? Did you (your patient) have another episode of AF that was confirmed by ECG Holter during the last 2 years? Did you (your patient) have any symptomatic episode of AF that was confirmed by ECG and led to a change or increase in chronic antiarrhythmic therapy during the last 2 years? A positive answer to at least one of the questions was considered as late AF recurrence.

### Blood Sampling

Blood sampling was performed after the signing of the informed consent in the morning prior to the CA. A sample of 5 ml of whole blood was collected in Sarstedt® Ethylene Diamine Tetraacetic Acid with potassium (EDTA-K) tubes; the blood was then processed immediately after collection in a pre-cooled centrifuge and was spun for 2000 *g* for 10 min at 4 °C. If the plasma was haemolytic after the centrifugation, novel blood sampling was performed. After centrifugation, only the upper portion of plasma was collected to avoid contamination of the sample with platelets, leukocytes and erythrocytes from the buffy coat, and the obtained plasma was aliquoted into Eppendorf tubes and frozen at − 80 °C until further processing.

### MicroRNA Discovery Pipeline and Validation

The study was divided into two distinct phases. In the first phase, exploration, an analysis of all miRNAs was performed using small RNA sequencing to identify novel miRNA candidates whose expression was different between the groups. The second phase, validation, was then conducted to validate the levels of the five most altered miRNAs in the rest of the cohort.

#### RNA Isolation

Total RNA enriched for small RNAs was isolated using an miRNeasy Serum/Plasma Kit® (QIAGEN, GmbH, Hilden, Germany) from the 250 µl of plasma. Samples were slowly defrosted on ice and then re-centrifuged at 1000*g* for 5 min at 4 °C to remove the remaining cellular components and other residues. After re-centrifugation, 200 µl of supernatant were collected for further processing and mixed with 750 μl of QIAzol Lysis Reagent and 1 μl glycogen solution (concentration 2 ng/μl; Sigma Aldrich, USA). After 5-min of incubation at room temperature, 200 µl of chloroform were added, and after another 2-min of incubation at room temperature, the mixture was centrifuged at 12,000 *g* for 15 min at 4 °C to induce phase separation – 500 µl of clear upper phase, containing RNA, were collected, and mixed with 750 µl of ethanol. The obtained mixture was centrifuged through the RNeasy Mini Spin Column. The filter within the Mini Spin Column was then washed using RWT and RPE Wash solutions and 80% ethanol to purify the RNA. Lastly, using 20 µl of DNAse/RNAse-Free Water, the RNA was extracted and frozen at − 80 °C until further processing.

#### Exploratory Phase: Small RNA Sequencing

From the whole cohort of 49 patients, ten body mass index (BMI)-, age- and sex-matched patients were selected, including five patients with AF recurrence and five without AF recurrence. Within this exploratory cohort, miRNA profiling was performed using small RNA next-generation sequencing (NSG) using the Illumina instrument (Illumina, San Diego, CA, USA). Preparation of complementary DNA (cDNA) libraries was performed using the CleanTag® Small RNA Library Prep Kit (Trilink, San Diego, USA). The quantity of cDNA libraries was measured using the Qubit and Qubit double strain DNA (dsDNA) High Sensitivity (HS) Assay Kit (Thermo Fisher Scientific, Waltham, MA, USA), and the length of cDNA libraries was measured using TapeStation Instrument 2000 and High Sensitivity D1000 ScreenTape (Agilent Technologies, Santa Clara, CA). Sequencing was done using the NextSeq 500/550 High Output Kit v2 and NextSeq 500/550 instrument (Illumina, San Diego, CA, USA), with the sequencing setup of 75 single read cycles.

Sequencing data were processed using the online tool Chimira, and statistical analysis of sequencing data was performed using the Bioconductor tool and DESeq2 algorithm.

#### Validation Phase: qRT-PCR

The top 5 most differently expressed miRNAs between the groups with and without AF recurrence were selected for validation. These miRNAs had to meet the requirements of a log2 fold change lower than − 0.5 or higher than 0.5, minimum number of reads (BaseMean) > 50 and unadjusted *p* value lower than 0.05. Validation was performed using quantitative reverse transcription polymerase chain reaction (qRT-PCR).

A sample of 2 μl of isolated undiluted RNA eluate was reversely transcribed using the TaqMan™ miRNA Reverse Transcription Kit (Applied Biosystems, Foster City, CA, USA) to prepare the cDNA template. qRT-PCR was performed using TaqMan™ miRNA Assays (Applied Biosystems, Foster City, CA, USA) according to the manufacturer’s instructions, using the Quant Studio 12K Flex System (Applied Biosystems, Foster City, CA, USA). The following miRNA assays were used: hsa-miR-190b (ID: 002263), hsa-miR-206 (ID: 000510), hsa-miR-326 (ID: 000542), hsa-miR-505-5p (ID: 002087) and mml-miR-1296-5p (475233_mat). All measurements were performed in duplicates (Supplementary Table 1, see the electronic supplementary material). Expression levels measured by qRT-PCR were normalized using two distinct approaches: firstly, using hsa-miR-16-5p as an endogenous control and formula (normalized expression) = 2^-(CtΔ miRNA duplicate-CtΔ miR-16-5p duplicate)^; secondly, using the overall number of PCR cycles (i.e. 40), using formula (normalized expression) = 2^-(CtΔ miRNA duplicate-40^^)^. Differences with unadjusted *p* < 0.05 or adjusted *p* < 0.1 were considered statistically significant.

#### Statistics

Statistical analysis was performed by the Department of Biostatistics of St. Anne’s University Hospital in Brno – International Clinical Research Centre (FNUSA-ICRC). Input data consisted of patient characteristics, data about the performed CA procedure, data about AF recurrence during the follow-up period, the levels of the five measured miRNAs normalized to expression of miR-16 or cycle 40 threshold value (Ct-40). Altogether, 31 continuous variables and nine categorical variables were evaluated. For descriptive purposes, standard parameters were used, such as mean, standard deviation (SD), minimum, maximum and quartiles.

Distributions of variables between the groups with early AF recurrence (at 6 months) and self-reported late AF recurrence (at 24 months) were processed. In the case of continuous variables, the Mann-Whitney test was used. In the case of categorical variables, the Fisher's exact test was applied. A similar approach was used in the comparison of patients with paroxysmal and persistent AF. The output from Mann-Whitney tests is both the classic *p* value for evaluated parameters and its adjusted value using the Benjamini-Hochberg correction for compensation for the error from multiple testing.

To study the relationship between identified miRNAs and AF recurrence, binary logistic regression modelling was performed. The dependent variable was the presence of AF recurrence (yes x no), and the independent variable was the plasmatic level of significantly altered miRNAs. Models were adjusted for age and BMI.

## Results

### Study Cohort Characteristics and Intergroup Comparison

Study cohort characteristics are provided in Table 1. Our cohort included 14 women and 35 men. Their average age was 63.14 ± 10.76 years at the date of procedure. Their average height was 174.45 ± 9.89 cm and weight 93.67 ± 17.23 kg (average BMI 30.74 ± 5.09 kg/m^2^). Within the study group, there were 24 patients with paroxysmal AF and 25 patients with persistent AF. From the past medical history, we identified 34 patients with arterial hypertension, 28 patients with hyperlipoproteinaemia, eight patients with type 2 diabetes mellitus and six patients with recorded coronary artery disease. Seven patients were current smokers. Patients with and without AF early recurrence at 6 months did not differ in the baseline characteristics or comorbidities; the only difference was observed in the ratio of persistent versus paroxysmal AF, with persistent AF being more common in patients with early AF recurrence.

**Table 1:** Study cohort characteristics

Parameter	All	No AF recurrence	Early AF recurrence	*P* value*
Number of patients	49	32	16	–
Men/women	35/14	23/9	11/5	–
Age (years)	63.14 ± 10.76	63.41 ± 10.75	62.00 ± 11.13	0.69
Height (cm)	174.45 ± 9.89	175.19 ± 9.55	173.44 ± 10.89	0.61
Weight (kg)	93.67 ± 17.23	91.97 ± 14.32	98.25 ± 21.78	0.31
BMI (kg/m^2^)	30.74 ± 5.09	29.89 ± 3.53	32.67 ± 7.11	0.20
Paroxysmal/persistent	24/25	20/12	3/13	**0.01**
Hypertension	34 (69.4%)	21	12	0.74
Diabetes mellitus	8 (16.3%)	6	1	0.40
Coronary disease	6 (12.2%)	4	2	˃ 0.99
Dyslipidaemia	28 (57.1%)	17	11	0.36
Current smokers	7 (14.3%)	4	3	0.91

*AF* atrial fibrillation, *BMI* body mass index

*P value for comparison of early AF recurrence and no AF recurrence

Patients with paroxysmal and persistent AF differed in BMI (29.20 ± 3.87 vs. 32.21 ± 5.72, *p* = 0.042), which was higher in the persistent AF group. Patients with persistent AF also presented with higher ablation time during the CA (2886.80 ± 1011.13 vs. 2245.92 ± 1127.75 second, *p* < 0.001) and higher ablation-points count (53.16 ± 19.58 vs. 43.63 ± 20.47, *p* < 0.001).

### Next-Generation Sequencing Reveals Several miRNAs to Differ Between Patients With and Without AF Recurrence

Altogether, NGS miRNA profiling revealed 1752 distinct miRNAs. All these miRNAs were compared between the patients with and without AF recurrence using the DESeq2 algorithm. Using unadjusted *p* value < 0.05, 53 distinct miRNAs differed between the groups. To select the most appropriate candidates, we applied the selection criteria of log2 fold change higher than 0.5 or lower than − 0.5 (to ensure biological relevance) and total number of reads above 50 (to avoid miRNAs with low abundance in the samples). After applying all these criteria, 22 miRNAs were shown to be different between the groups, as summarized in Table 2.

**Table 2:** Plasmatic microRNAs altered between patients with and without AF recurrence at 6 months

miRNA number	Number of reads	Log2 fold change	*P* value
hsa-miR-190b	174	0.99	0.008
hsa-miR-1296-5p	74	− 0.81	0.010
hsa-miR-505-5p	68	1.29	0.011
hsa-miR-326	981	− 0.87	0.012
hsa-miR-206	187	2.12	0.014
hsa-miR-1294	59	1.25	0.019
hsa-miR-574-3p	618	1.12	0.021
hsa-miR-223-5p	3065	0.90	0.023
hsa-miR-1255b-5p	61	1.23	0.030
hsa-miR-29c-5p	166	−0.58	0.030
hsa-miR-4732-5p	153	1.39	0.031
hsa-miR-139-3p	714	1.04	0.032
hsa-miR-183-5p	574	1.41	0.035
hsa-miR-200c-3p	245	0.70	0.037
hsa-miR-182-5p	995	0.93	0.039
hsa-miR-4446-3p	146	1.04	0.039
hsa-miR-361-3p	1972	1.04	0.040
hsa-miR-766-3p	113	− 1.32	0.041
hsa-miR-93-5p	40373	0.55	0.042
hsa-miR-454-5p	105	− 0.59	0.046
hsa-miR-760	180	0.87	0.047
hsa-let-7d-3p	2351	0.55	0.049

*AF* atrial fibrillation

### Validation of Identified miRNA Levels Using qRT-PCR

#### hsa-miR-206 Levels are Increased in Patients with Early AF Recurrence

For the validation of NGS results, the top 5 most altered miRNAs were selected, including hsa-miR-190b, hsa-miR-1296-5p, hsa-miR-505-5p, hsa-miR-326 and hsa-miR-206. Analysis was performed in 48 patients, as one patient was lost to follow-up.

**Fig. 1:**
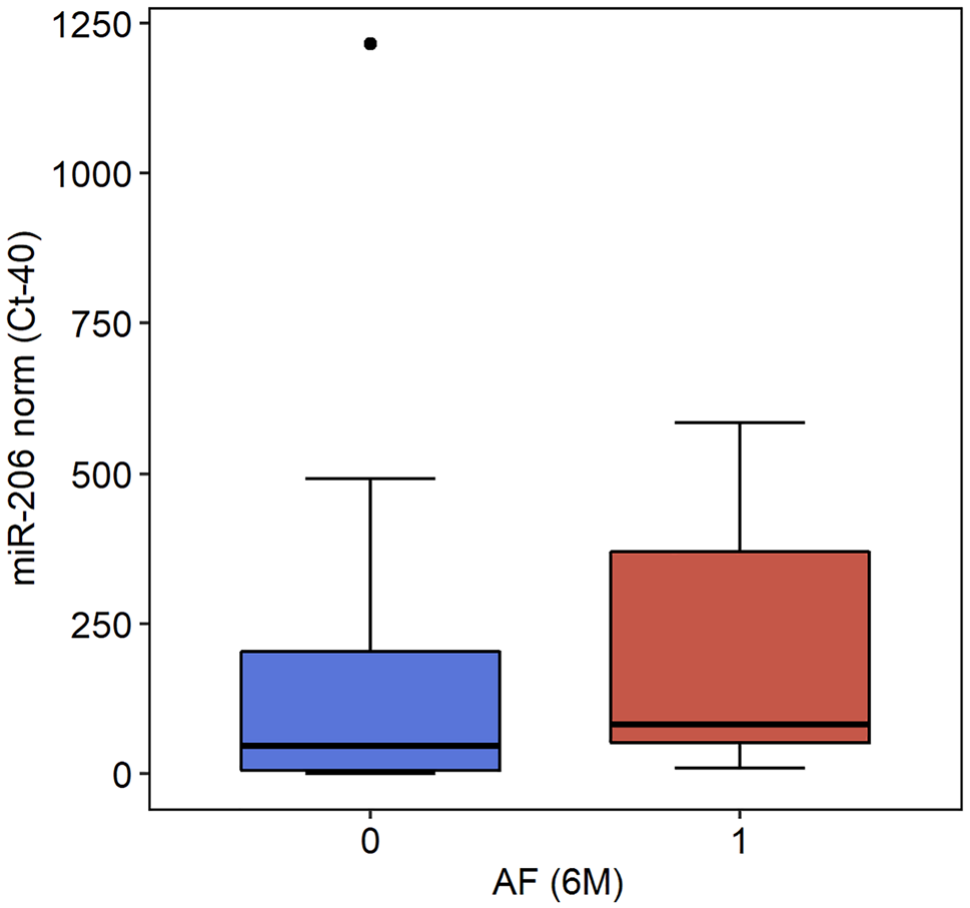
Relative miR-206 levels in patients with/without AF recurrence. The relative expression levels of hsa-miR-206 in patients without (left, 0) and with (right, 1) AF recurrence is shown. The standard box plots show the median (black line), interquartile range (box), minimum and maximum (lines) and outliers (dots). *AF* atrial fibrillation, *Ct-40* cycle 40 threshold value

We observed a statistically significant increase in plasmatic levels of hsa-miR-206 (normalized to CT-40, *p* = 0.023, see Fig. 1) and an increase of borderline significance in miR-505-5p (normalized to CT-40, *p* = 0.090) and hsa-miR-206 normalized to miR-16 (*p* = 0.078) between the group with AF recurrence and the group without AF recurrence. After adjustment for multiple comparisons, the differences become insignificant.

#### miR-190b and miR-1296-5p Levels are Increased in Patients with Late AF Recurrence

The secondary aim of the study was to test whether the same miRNAs differ also between the patients with self-reported (patients were contacted remotely) late recurrence of AF. For self-reported late AF recurrence, significant differences in plasmatic levels of miR-190b (*p* = 0.013) and miR-1296-5p (*p* = 0.036), both normalized to CT-40, were observed. Similarly to previous results, after adjustment for multiple comparisons, the differences became insignificant.

#### Regression Modelling Confirms hsa-miR-206 as a Potential Predictive Biomarker of AF Recurrence

miRNA levels may be affected by the presence of comorbidities, by body composition or by age. To further study the relationship between significantly altered miRNA levels (hsa-miR-206 for early recurrence; hsa-miR-190b and hsa-miR-1296-5p for late recurrence), binary regression modelling was performed. Results from the models are summarized in Table 3.

**Table 3:** Binary logistic regression adjusted for BMI and age

Variable	OR (CI)	*P* value
**miR-206**	**2.65 (1.20–8.59)**	**0.049**
miR-190b	0.99 (0.97–1.00)	0.151
miR-1296-5p	1.00 (1.00–1.00)	0.182

*BMI* body mass index, *OR* odds ratio *CI* confidence interval

The model involving hsa-miR-206 (normalized to hsa-miR-16) showed statistical significance. Within the binary model, the dependent variable was the presence of AF recurrence (yes x no) and the independent variable was the plasmatic level of hsa-miR-206. The model was adjusted for age and BMI. For better interpretability, miR-206 plasmatic levels were multiplied by 1000. The multiplication was made because the difference of miR-206 levels was approximately 0.001 and the logistic regression counts with changes of 1 unit. From this modelling, we saw a 2.65 times higher chance of early AF recurrence related to an increase in plasmatic levels of hsa-miR-206 by 1 unit (*p* = 0.049). Receiver operating characteristics (ROC) were calculated, showing an area under the curve (AUC) of 0.679, and by using the cut-off of 0.5, we reached a sensitivity of 97%, specificity of 46%, model accuracy of 81% and balanced accuracy of 71%.

## Discussion

Recurrence of AF after CA still represents an important clinical issue, and various attempts have been tested regarding alleviating this phenomenon [20]. There are various clinical, laboratory and echocardiography markers known to be associated with AF recurrence, such as type of AF, age, sex, eGFR, presence of left ventricular systolic or diastolic dysfunction, left atrial mechanical dyssynchrony or functional mitral regurgitation [21–23]. Nevertheless, a specific and easily obtainable plasmatic marker to determine the risk of AF recurrence is still missing. Plasmatic miRNAs, together with other groups of non-coding RNAs, such as long non-coding RNAs (lncRNAs) [24], are emerging biomarkers in various fields of medicine, including the cardiovascular field. [25,26]

Firstly, there are studies that focused on the circulating miRNAs and their differences between healthy controls and patients with AF, then there are studies focused on miRNA changes among various types of AF and, lastly, there are studies focused on miRNAs altered before and after CA. Selection of miRNAs in this first group of studies was based on knowledge of AF pathophysiology, determining the miRNAs known to be involved either in the initiation of AF and/or miRNAs involved in its persistence and after-effects, such as myocardial remodelling or fibrosis [18]. Various miRNAs, such as miR-4443 [27] or miR-4798-3p [28], were identified to be altered between patients with and without AF, while miRNAs such as miR-21 [11,29,30], pri-miR-126 [31], miR-150 [11,29], miR-409-3p and miR-432 [32] were tested and their levels were shown to be altered before and after CA or between patients with different types of AF. Results of mentioned studies have recently been reviewed in the meta-analysis by Menezes et al. [33]; nevertheless, even the meta-analysis shows that results of the individual studies are conflicting, e.g. higher levels of fibrosis-related miR-21 showed either a decreased [11,29] or increased [30] chance of AF recurrence after CA.

The mentioned approach, i.e. identifying novel candidate miRNAs among those related to the pathophysiology of AF, is partially limited, as miRNAs with no relation to the pathophysiology of AF, but with promising biomarker potential, may be missed. Thus, a second approach to identify novel miRNA biomarkers is the use of novel high-throughput methods, such as small RNA sequencing. This approach was applied in our study, and we have identified 22 possibly altered miRNAs, among which we have confirmed hsa-miR-206 to be increased in the plasma of patients with early AF recurrence, and in regression modelling, we showed that an increase in miR-206 levels by 1 unit increases the risk of AF recurrence after CA by 2.65 times. The only study that used NGS for identification of AF recurrence-related plasmatic miRNAs was done by Kiliszek et al. in 2020 [19]. The authors used similar methodology and cohort to our study and revealed 34 miRNAs to be altered between patients with and without AF recurrence after CA [19]. Similarly to our study, there was no overlap with the previously published studies focusing on miR-21 [11,29,30], pri-miR-126 [31], miR-150 [11,29], miR-409-3p and miR-432 [32], and when compared to our findings, there is an overlap in five miRNAs (hsa-miR-183-5p, hsa-miR-182-5p, hsa-miR-574-3p, hsa-miR-326, hsa-miR-1294) when using data from the NGS exploration of both studies. Comparison of qRT-PCR validation results is more complicated, as Kiliszek et al. did not confirm any plasmatic miRNA changes identified during NGS by qRT-PCR and focused mainly on miRNAs not identified in our study. Only in the case of hsa-miR-326 can we confirm their negative results, as this miRNA did not differ between the patient groups in our study. Unfortunately, hsa-miR-206, identified in our study, was not detected by Kiliszek et al., and other miRNAs overlapping in NGS results (hsa-miR-183-5p, hsa-miR-182-5p, hsa-miR-574-3p, hsa-miR-1294) were not tested in our study due to the limited sample volumes and economic costs, as we focused mainly on the top 5 altered miRNAs from our NGS results list.

Despite not being considered a myocardial-specific miRNA, hsa-miR-206 is known as a potential prognostic marker in various cancers [34] and is proposed as a skeletal muscle-“specific” miRNA in already published studies; however, these results are true only under physiological conditions [35]. Animal studies showed the relation of miR-206 with the pathophysiology of cardiac arrhythmias by downregulating Connexin43 – a cardiac gap junction protein, known to be involved also in the pathophysiology of AF [36]. Similarly, in a canine study, the authors linked miR-206 to the initiation of AF by targeting the gene for GTP cyclohydrolase I, the enzyme responsible for control of tetrahydrobiopterin synthesis. With a higher level of GTP cyclohydrolase I, the cardiac autonomic nervous remodelling was inhibited, and it lowered the initiation of AF [37]. The same results on miR-206’s influence on cardiac autonomic nervous remodelling were observed in another canine study, but the authors pointed to a different pathway by targeting superoxide dismutase 1 [38]. Thus, despite being more muscle specific than myocardium specific under physiological conditions, during the pathogenesis of AF, its levels were shown to be altered even in the myocardium, thus potentially explaining its possible involvement in predicting AF recurrence after CA in our study.

Low reproducibility and inter-comparability of plasmatic miRNA biomarker studies is a pressing issue in the field. Studies focusing on a similar topic are usually slightly different in their specific aims and inclusion and/or exclusion criteria; they also differ in sample sizes and used methodologies ranging from pre-analytic processing through RNA isolation to qRT-PCR validation. In the case of a complex disease such as AF, this may then lead to observed contradicting results that shall be explained by further larger scale, properly power-sized and multicentric trials using standardized operating procedures for all steps of RNA biomarker discovery. Use of such standardized operating procedures is one of the strengths of our study, together with the use of adequate statistical methods; nevertheless, the limitations of our study are its sample size and inability to validate more than the top 5 altered miRNAs due to the economic issues and low RNA sample volumes.

## Conclusions

In our study, we identified a potential set of 22 miRNAs whose levels are altered between patients with and without AF recurrence after CA, using small RNA sequencing. Of these, we validated the plasmatic levels of five miRNAs using qRT-PCR, and only the levels of hsa-miR-206 differed significantly between the groups. In the binary logistic regression modelling with the adjustment for age and BMI, higher plasma hsa-miR-206 levels predicted AF recurrence (2.65 times higher odds for AF recurrence with the increase of miR-206 by 1 unit). Larger scale studies shall be performed to confirm our results, together with the results of other authors^11,19,29–32^, to identify reliable and clinically relevant miRNA-related markers of AF recurrence.

## Supplementary Information

Below is the link to the electronic supplementary material.

Supplementary file1 (DOCX 20 KB)

## Declarations

### Data Availability

The data from RNA sequencing are available on request with the authors.
