# Supplementary material for: Identification of Plasmatic MicroRNA-206 as New Predictor of Early Recurrence of Atrial Fibrillation After Catheter Ablation Using Next-generation Sequencing
Source: Mol Diagn Ther. 2024 Mar 8;28(3):301–10. doi: 10.1007/s40291-024-00698-x (PMC11068688; doi:10.1007/s40291-024-00698-x)
Supplement: Supplementary file 1 — Supplementary file1 (DOCX 20 KB) [file 40291_2024_698_MOESM1_ESM.docx]

**Supplementary Table 1**: Mean CT values for 5 identified miRNAs

| Patient No | **miR-190b CT** | **miR-206 CT** | **miR-326 CT** | **miR-505-5p CT** | **miR-1296-5p CT** |
| --- | --- | --- | --- | --- | --- |
| 1 | 33,541 | 29,753 | 27,039 | 26,949 | 26,167 |
| 2 | 40 | 31,991 | 40 | 25,021 | 33,895 |
| 3 | 32,847 | 31,468 | 26,378 | 26,526 | 25,486 |
| 4 | 33,633 | 37,413 | 30,366 | 28,429 | 28,242 |
| 5 | 32,368 | 33,521 | 29,698 | 25,871 | 26,009 |
| 6 | 35,646 | 35,398 | 30,129 | 28,527 | 28,725 |
| 7 | 34,989 | 31,906 | 30,126 | 27,945 | 27,473 |
| 8 | 34,900 | 36,675 | 29,826 | 27,682 | 27,393 |
| 9 | 40,000 | 34,528 | 36,508 | 27,474 | 29,968 |
| 10 | 31,780 | 29,512 | 25,216 | 25,650 | 23,996 |
| 11 | 33,008 | 32,791 | 26,983 | 26,249 | 25,881 |
| 12 | 37,308 | 30,961 | 33,059 | 28,347 | 34,027 |
| 13 | 33,989 | 32,458 | 29,668 | 27,899 | 27,100 |
| 14 | 33,675 | 39,117 | 27,256 | 26,506 | 26,430 |
| 15 | 32,800 | 34,253 | 26,760 | 26,684 | 25,554 |
| 16 | 32,700 | 31,626 | 27,988 | 27,344 | 27,148 |
| 17 | 33,329 | 31,060 | 28,492 | 26,898 | 26,378 |
| 18 | 33,621 | 37,163 | 26,372 | 26,413 | 25,569 |
| 19 | 35,169 | 33,631 | 30,098 | 27,263 | 27,370 |
| 20 | 35,610 | 33,412 | 31,153 | 29,438 | 29,025 |
| 21 | 35,562 | 33,969 | 32,122 | 30,872 | 30,270 |
| 22 | 38,188 | 38,148 | 37,369 | 30,024 | 32,145 |
| 23 | 37,099 | 38,129 | 32,372 | 29,865 | 29,991 |
| 24 | 40,000 | 30,809 | 40,000 | 25,902 | 37,388 |
| 25 | 34,537 | 34,195 | 29,862 | 27,721 | 26,424 |
| 26 | 34,953 | 31,134 | 28,541 | 26,681 | 26,099 |
| 27 | 35,398 | 37,867 | 29,981 | 27,648 | 27,948 |
| 28 | 34,798 | 33,698 | 28,122 | 26,942 | 26,931 |
| 29 | 34,068 | 36,406 | 29,146 | 27,170 | 27,282 |
| 30 | 37,104 | 30,896 | 34,714 | 34,198 | 36,516 |
| 31 | 36,568 | 35,671 | 34,703 | 31,211 | 40,000 |
| 32 | 28,559 | 31,939 | 27,543 | 24,052 | 25,039 |
| 33 | 36,500 | 37,128 | 31,988 | 32,636 | 30,838 |
| 34 | 36,096 | 40,000 | 30,928 | 29,880 | 30,126 |
| 35 | 35,304 | 40,000 | 32,733 | 29,807 | 32,968 |
| 36 | 34,807 | 32,606 | 29,248 | 27,830 | 27,728 |
| 37 | 34,248 | 31,792 | 29,727 | 27,147 | 27,250 |
| 38 | 33,789 | 32,285 | 28,134 | 27,870 | 26,789 |
| 39 | 33,769 | 34,743 | 27,904 | 27,540 | 26,019 |
| 40 | 36,478 | 34,293 | 30,877 | 28,650 | 29,524 |
| 41 | 34,603 | 36,046 | 30,051 | 27,064 | 27,423 |
| 42 | 33,223 | 32,248 | 27,276 | 25,963 | 25,252 |
| 43 | 33,439 | 33,671 | 28,135 | 26,507 | 26,032 |
| 44 | 34,694 | 31,537 | 29,820 | 28,764 | 27,781 |
| 45 | 33,269 | 34,189 | 29,037 | 27,493 | 27,272 |
| 46 | 33,904 | 32,714 | 28,172 | 26,008 | 25,965 |
| 47 | 34,045 | 34,447 | 27,763 | 26,907 | 25,993 |
| 48 | 33,728 | 38,371 | 27,420 | 27,114 | 26,498 |
| 49 | 35,785 | 30,879 | 32,273 | 26,427 | 26,035 |

**Caption**: Supplementary table showing the mean qRT-PCR results (CT values) for plasmatic miRNAs tested in validation phase.
